# Effect of water, sanitation and hygiene interventions on active trachoma in North and South Wollo zones of Amhara Region, Ethiopia: A Quasi-experimental study

**DOI:** 10.1371/journal.pntd.0006080

**Published:** 2017-11-10

**Authors:** Beselam Tadesse, Alemayehu Worku, Abera Kumie, Solomon Abebe Yimer

**Affiliations:** 1 Ethiopian Institute of Water Resources (EIWR), Addis Ababa University, Addis Ababa, Ethiopia; 2 Amhara National Regional State, Water, Irrigation and Energy Development Bureau, Bahir Dar, Ethiopia; 3 Addis Ababa University, College of Health Sciences, School of Public Health, Addis Ababa, Ethiopia; 4 Department of Microbiology, Oslo University Hospital, Oslo, Norway; Institut Pasteur, FRANCE

## Abstract

**Background:**

Trachoma is chronic kerato conjunctivitis, which is caused by repeated infection with *Chlamydia trachomatis* bacterium. It is hyper endemic in many rural areas of Ethiopia. The objective of this study was to measure the effect of water, sanitation and hygiene interventions on active trachoma in selected woredas of North and South Wollo zones of Amhara Region, Ethiopia.

**Methodology:**

A community based quasi-experimental study was conducted from October 2014 to December 2015 among children aged 1–8 years at baseline and among one year older same children after intervention. A four-stage random cluster-sampling technique was employed to select study participants. From each selected household, one child was clinically assessed for active trachoma. Structured questionnaire was used to collect socio demographic and behavioral data. MacNemar test was applied to compare the prevalence of active trachoma between baseline and after the intervention period at both intervention and non-intervention study areas.

**Results:**

The prevalence of active trachoma was reduced from baseline prevalence of 26% to 18% after one-year intervention period in the intervention woredas (*P*≤0.001). MacNemar test result showed significant reduction of active trachoma prevalence after the intervention period in the intervention woredas compared to the non-intervention woredas (P≤0.001). Water, sanitation and hygiene related activities were significantly improved after the intervention period in the intervention woredas (P<0.05).

**Conclusions:**

There was a significant reduction of active trachoma prevalence between the baseline and after the intervention period in the intervention woredas, but not in the non-intervention ones. Improved water, sanitation and hygiene interventions contributed to the reduction of active trachoma. However, the magnitude of active trachoma prevalence observed after the intervention is still very high in the studied areas of North and South Wollo Zones communities. To achieve the global trachoma elimination target by the year 2020 as set by the WHO, continued WaSH interventions and periodic monitoring, evaluation and reporting of the impact of WaSH on active trachoma is warranted.

## Author summary

Trachoma is an infectious disease of the eye, which is caused by repeated infection with Chlamydia trachomatis bacterium. The disease is the leading cause of preventable blindness. Ethiopia is the most trachoma affected country in the world. The World Health Organization (WHO) recommends Water, Sanitation and Hygiene (WaSH) interventions to control and eliminate blinding trachoma. With the aim of assessing the effect of WaSH intervention on active trachoma in selected woredas/districts of North and South Wollo zones of Amhara Region, Ethiopia, we selected intervention and control woredas, and WaSH program was implemented in the intervention woredas for one year. Baseline trachoma prevalence was conducted in both intervention and control areas before WaSH program was implemented. Prevalence was determined in both study areas after one-year intervention period and was compared with the baseline. The finding showed that the prevalence of active trachoma was reduced from baseline prevalence of 26% to 18% after one-year WaSH program implementation in the intervention woredas compared to the non-intervention woredas. However, as the magnitude of active trachoma prevalence observed after the intervention is still very high. Continued WaSH interventions and periodic monitoring, evaluation and reporting of the impact of WaSH on active trachoma is warranted in the studied areas of Ethiopia.

## Introduction

Trachoma is a chronic kerato conjunctivitis, which is caused by repeated infection with *Chlamydia trachomatis* bacterium. The disease is often transmitted by fomites (objects or materials that are likely to carry infectious organisms), contaminated fingers with discharge from the eyes or nose of an infected person, and through eye-seeking fly *Musca sorbens* [1]. Trachoma is the leading cause of infectious blindness in the world [2]. Globally, trachoma is responsible for the visual impairment of about 2.2 million people, of whom 1.2 million became irreversibly blind [3].

Ethiopia is one of the five countries in the world where half of the global burden of active trachoma is concentrated [4]. Of the ten National Regional States in Ethiopia, the Amhara National Regional State (ANRS) is disproportionately affected by trachoma [4]. A former study indicated a 52% and 13% overall prevalence of trachomatis inflammation-follicular (TF) among children aged 1–9 years in North and South Wollo zones of Ethiopia, respectively [5].

Evidence show that some improvements in the control of trachoma have been brought through socioeconomic development and control programs along with other disease control programs in many countries. However, trachoma continues to be hyperendemic in many of the poorest rural areas of the world, especially in areas that have limited access to water and sanitation [2].

The prevention, control and eventual elimination of trachoma depend heavily on the availability of improved Wash programs in endemic communities [6]. As antibiotic treatment alone will not break the cycle of transmission, improvements of WaSH infrastructure and appropriate health seeking behavior of patients are essential to achieving sustained control, elimination, or eradication of trachoma [7–9].

To achieve the elimination goal of blinding trachoma as a public health problem, the World Health Organization (WHO) endorsed the SAFE strategy (Surgery for correction of trichiasis; Antibiotics to treat infection; Facial cleanliness and Environmental improvement to reduce chlamydia transmission) have been implemented by different stakeholders in ANRS for the last 10 years. As reported by the Carter Center, Ethiopia, the “S” and “A” components of the SAFE strategy were the most implemented ones in the region [10].

Majority of the previous trachoma studies conducted in Ethiopia focused on analyzing the magnitude and associated factors of the disease. Consequently, programs were designed and predominantly implemented in the most severely trachoma affected areas based on SAFE strategy. However, there are limited studies regarding the effect of each component of the SAFE strategy on trachoma control. Therefore, this study aimed at examining the effect of WaSH interventions on active trachoma elimination in North and South Wollo Zones of Amhara Region, Ethiopia. We conducted a baseline active trachoma prevalence survey in selected woredas in 2014. The objective of the current study was to measure the effect of WaSH program implementation on active trachoma burden after one year intervention period in the same woredas where the base line survey was conducted.

## Methods and materials

### Ethics statement

This study was approved by Ethical Review Committee of Ethiopian Institute of Water Resources. Permission was also obtained from the ANRS and the District Health Offices to conduct the study. In addition, *Kebele* Administrators were informed about the purpose of the study. Before the commencement of data collection, caregivers of a child were adequately informed by data collectors about the objectives and importance of their involvement in the study, the confidentiality of the information they provided, the time that the interview will take and other relevant information. Finally, caregivers of a child who were willing to take part in the study provided written informed consent.

### Study setting

The study was conducted in North and South Wollo Zones of ANRS, Ethiopia. Based on the Federal Democratic Republic of Ethiopia Central Statistical Agency population projections, North Wollo Zone has a total estimated population of 1,764,655. Majority (86%) of the population of the zone are rural inhabitants. Eighty three percent of the population practiced Orthodox Christianity and the remaining 17% were Muslims. South Wollo Zone has a total estimated population of 2,980,618, and (84%) of the people reside in rural areas. Muslims and Orthodox Christians comprised 71% and 29% of the total population of the zone, respectively [11].

The WHO recommended WaSH program is being implemented in selected woredas of ANRS. Considerable proportion of woredas in North and South Wollo Zones are included in this initiative. The main objective of the WaSH program is to increase access to improved water supply and sanitation services for inhabitants in participating woredas/towns in ANRS and Ethiopia at large. Similarly, the Amhara Trachoma Control Program (ATCP) has been operating in Dessie Zuriya and Raya Kobo woredas of North and South Wollo zones. The program’s overall objective complements the WHO campaign for the global elimination of blinding trachoma by the year 2020 and The ATCP adopts multi-sectoral comprehensive approach. Provision of water and hygiene and sanitation education have been the focus areas of the program.

A total of 177 improved water supply schemes were constructed in Raya Kobo and Dessie Zuriya Woredas after the WaSH program was introduced. The water supply schemes were thus increased to 508 and 387 in Raya Kobo and Dessie Zuriya Woredas from the previous numbers of 406 and 312, respectively [12]. Among the 177 water schemes developed, 37 were constructed by the ATCP within the one-year project period (May 2014-July 2015) [13]. Improved latrine coverage has increased to 70% and 79% in Raya Kobo and Dessie Zuriya Woredas from the previous percentage of 64% and 67%, respectively after the intervention period [14]. In addition, within the one-year project period of ATCP, two model latrines were constructed in intervention Woredas in public places close to health centers. Moreover, the community sensitization activities by ATCP workers have improved the construction and utilization of latrines [15].

Health education was provided through the establishment of Anti-Trachoma School Clubs in primary schools and through sensitization of the community by village hygiene educators. There were classroom trachoma ambassadors and sanitation corners in school compounds. These ambassadors check the personal hygiene situation of each student every morning at the time of flag ceremony. Students found with poor personal hygiene were taken to the sanitation corner to wash his/her hand/face and get health education about the role of hygiene on health, specifically on trachoma. The Anti-Trachoma School Clubs conducted trachoma prevention sensitization activities in the community at public gatherings. This helped to raise awareness of trachoma prevention and to promote the construction and use of latrines at household level. Community level uptake of latrines and other environmental improvements was monitored at school level with students’ under taking hygiene and sanitation audits.

Village hygiene educators as part of the WaSH committee promoted the effective use of water for face washing and other hygiene practices, and for the development and uptake of improved sanitation through house to house visits and participating community meetings. Health extension workers and village hygiene educators worked together on the construction and utilization of improved latrines, solid and liquid waste disposals and other hygiene and sanitation related activities.

In the non-intervention woredas (Gubalafto and Tehuledere) a total of additional 101 improved water supply schemes were constructed from October 2014 to September 2015 [12]. The latrine coverage was improved to 30% and 88% in Gubalafto and Tehuledere woredas from the previous percent of 25% and 84%, respectively [14]. In both intervention and non-intervention woredas, all the respective organizations were implementing the same activities however, additional water supply schemes and sanitation services were constructed by the ATCP in the intervention woredas.

### Design, sample size and sampling methods

This was a quasi-experimental study. Sample size was calculated using Epi-Info software package. Therefore, by considering a 95% confidence interval (Z_α/2_ = 1.96), 5% for type I error, 80% power, design effect of 1.5, 52% prevalence of active trachoma from a previous study [6], a 42% proportion of active trachoma at some future date such that the quantity of (p_2_-p_1_) would be the size of the magnitude of change (10% difference), and a 10% non-response rate, the total sample size was calculated to be 1358 participants.

**Fig 1:**
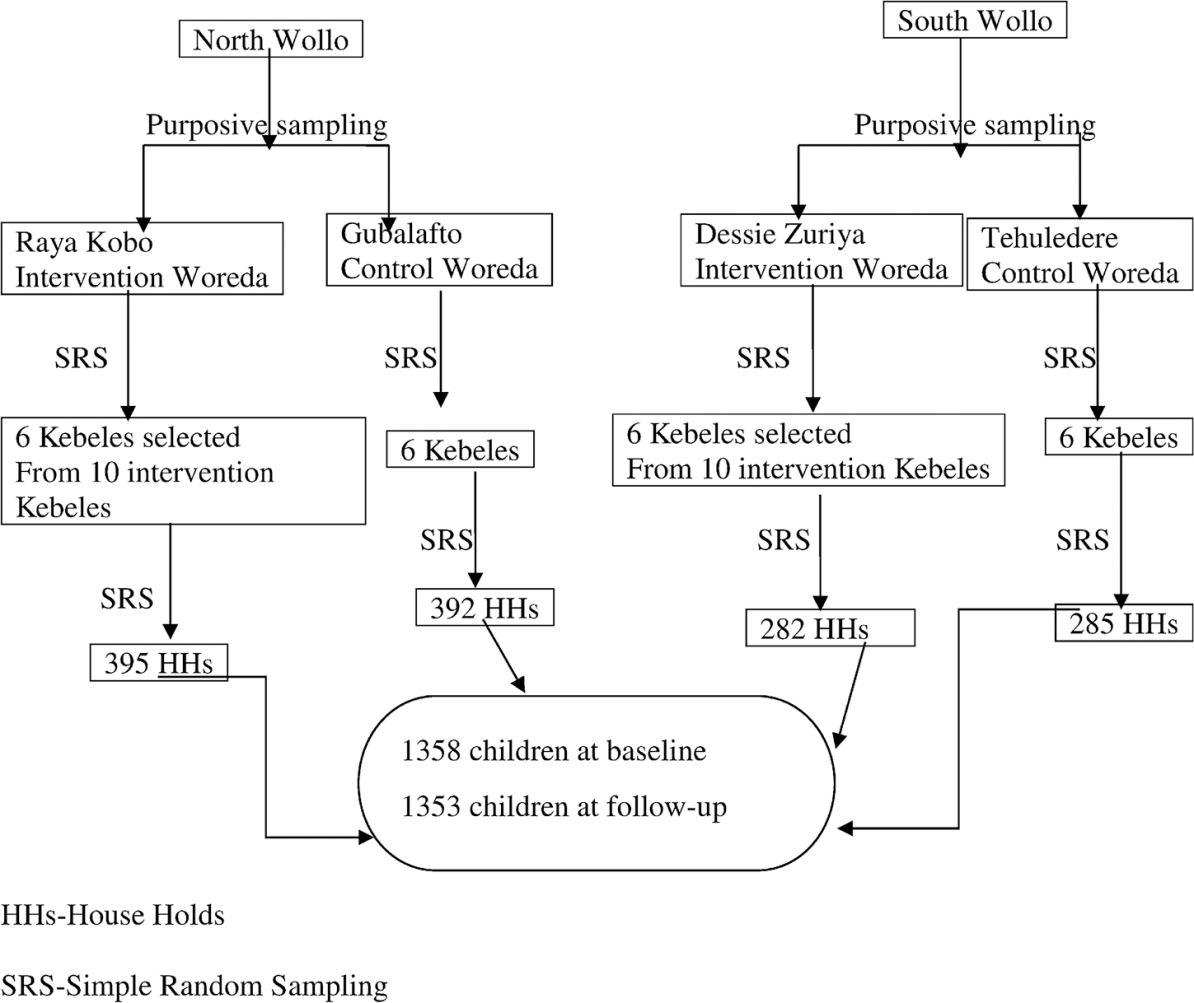
Sampling procedure for selecting the study units and participants.

A four-stage random cluster-sampling technique was employed for selecting the study units and participants. In the first stage, four woredas were purposively selected by taking Raya Kobo and Gubalafto woredas from North Wollo, and Dessie Zuriya and Tehuledere woredas from South Wollo Zones. The presence or absence of ATCP water provision and health education on hygiene and sanitation intervention was used as criteria for selecting and including woredas in the study. Accordingly, Raya Kobo and Dessie Zuriya woredas were already selected as intervention woredas by the WaSH program implementers. Simultaneously, non-WaSH intervention woredas (Gubalafto and Tehuledere woredas) were selected as control group by the research team for comparison purposes. In the second stage, six *kebeles* (smallest administrative units) were randomly selected from each intervention and non-intervention woredas. The six *kebeles* in the intervention woredas were randomly selected out of the ten intervention *kebeles*. In total, 24 rural *kebeles* were included in the study. In the third stage of the selection process, households were randomly selected from each selected nominated *kebele*. The numbers of households in each woreda were decided based on the woreda level population proportion of children. In the fourth stage of sampling, only one child was randomly selected from each selected household to participate in the study. Those households that did not have eligible children were excluded from the study and were replaced by another household [Fig 1.].

### Baseline survey

At baseline, children aged 1–8 years were randomly selected from each randomly selected household. This survey was conducted from October to December 2014 and recently published [16].

### Follow up survey

This survey took place from October to December 2015, 12 months after the baseline survey was conducted. It was done among the same children examined at baseline following similar survey methods.

### Data collection

A semi-structured questionnaire was used to collect data. The data collectors were four university graduates and were adequately trained regarding the data collection process. The questionnaire was pre–tested in a non-study area and the necessary corrections were made before the actual data collection was commenced. Collected data included socio demographic, environmental and behavioral factors of active trachoma. Behavioral factors such as face and hand washing practices were measured by asking the caregivers of a child using a structured questionnaire. Information on variables such as primary source of water, amount of water consumed per day, distance to water source and availability of latrine facilities were collected from caregivers of diagnosed children for trachoma. The volume of water consumed per day was calculated by estimating the volume of fetched water per day and dividing it by the number of family members. A round trip distance to water source was measured in hour. In addition, data for WaSH practice were collected from caregivers of diagnosed children for trachoma.

Clinical eye examination for the presence or absence of active trachoma was performed for each child participating in the study. Two nurses who participated as eye examiner in the previously conducted zonal trachoma survey took part in clinical diagnosis of active trachoma among the study participants. Simultaneously each child’s face was examined for facial cleanliness by the nurses and the presence or absence of ocular/nasal discharge was properly recorded. The clinical examiners didn’t have any information about those intervention and control woredas (they were blinded). In addition, they were not informed about the follow up survey.

Binocular loupes manufactured by Donegan optical company, Inc. at USA (×2.5 magnifications) and penlight torches were used during eye examinations. The right eyes followed by the left were examined to avoid failure to recall in which eye the examiner saw an abnormality. Then, the diagnosed children were classified according to WHO simplified trachoma-grading card as trachomatous inflammation-follicular (TF), trachomatous inflammation-intense (TI), and trachomatous scarring (TS) or free from trachoma [17]. All trachoma-positive individuals were treated with topical tetracycline eye ointment and were advised to consult the nearby health center for further eye health follow-ups. The principal investigator and the supervisors closely monitored the entire data collection processes. The filled-out questionnaires and eye examination results were collected and delivered to supervisors after checking for consistency and completeness on daily bases.

### Operational definition

**Improved water source-**is a water source protected by construction from outside contamination

**Improved latrine**-is well functioning latrine with locally made roofs, walls, floors and toilet seats.

**Unimproved water source-**is a water source unprotected by construction from outside contamination.

**Woreda**-refers to the third-level administrative entity of Ethiopia.

### Data quality assurance

In this study, several data quality assurance methods were used. Trainings and field guides were given to data collectors and supervisors. Pretests were made in a non-study area before the actual data collection was started. Intensive supervision was conducted throughout the survey period by trained supervisors and the principal investigator. Questionnaires were checked for consistency and completeness by supervisors at the end of each day.

### Data management and analysis

The raw data were entered using Epi Info Version 3.5.1 and exported to Statistical Package for Social Sciences (SPSS) IBM Version 20 (SPSS Inc. Chicago, IL, USA) for analysis. The analysis part contains descriptive statistics (frequency, percentage, mean and standard deviation). The prevalence’s of active trachoma between baseline and follow up surveys were compared both in the intervention and non-intervention groups using the MacNemar test. In addition, MacNemar test was used to compare the status of water, sanitation and hygiene related activities between baseline and follow up surveys.

## Results

### Characteristics of the study households and participants

At baseline, 1,358 children (1–8 years of age) were examined for active trachoma and were followed for one year. After one-year intervention period, 1,353 of the same children who were included in the baseline survey were examined for active trachoma. The socio demographic characteristics of the study participants were almost the same before and after the intervention period. Majority (96%) of the participants in both intervention and non-intervention woredas were married. Regarding their religion, almost half of them were Orthodox Christians. On average, 55% of heads of the households in both intervention and control woredas were illiterate, while 45% attended primary school and above (Table 1).

**Table 1:** Socio-demographic characteristics of study participants in the study woredas of North and South Wollo Zones, ANRS, Ethiopia, 2014.

Characteristics	Baseline (n = 1358)				After intervention (n = 1353)			
	Intervention Woredas (n = 678)		Non-Intervention Woredas (n = 680)		Intervention Woredas (678)		Non-Intervention Woredas (675)	
	Frequency	%	Frequency	%	Frequency	%	Frequency	%
Primary care giver of a child								
Mother	633	93.4	654	96.2	646	95.3	651	96.4
Grandmother	45	6.6	26	3.8	32	4.7	24	3.6
Sex of head of household								
Male	451	66.5	419	61.6	451	66.5	413	61.2
Female	227	33.5	261	38.4	227	33.5	262	38.8
Marital status of head of household								
Married	648	95.6	651	95.7	648	95.6	647	95.9
Divorced	23	3.4	24	3.5	23	3.4	23	3.4
Widowed	7	1.00	5	0.8	7	1	5	0.7
Religion of head of household								
Orthodox Christian	362	53.4	374	55	362	53.4	373	55.3
Muslim	316	46.6	306	45	316	46.6	302	44.7
Age of head of household (years)								
18–29	371	54.7	321	47.2	371	54.7	319	47.3
30–44	256	37.8	300	44.1	256	37.8	298	44.1
45–59	40	5.9	54	7.9	40	5.9	54	8.0
60+	11	1.6	5	0.8	11	1.6	4	0.6
Education status of head of household								
Illiterate	438	64.6	303	44.6	438	64.6	301	44.6
Literate	240	35.4	377	55.4	240	35.4	374	55.4
Heads of household primary occupation								
Farming and/or cattle rearing	647	95.4	638	93.8	647	95.4	633	93.8
Employee	18	2.7	17	2.5	18	2.7	17	2.5
Trade	10	1.5	21	3.1	10	1.5	21	3.1
Daily laborer	3	0.4	4	0.6	3	0.4	4	0.6
Family size								
1–3	426	62.8	440	64.7	426	62.8	436	64.6
4–6	192	28.3	180	26.5	192	28.3	179	26.5
≥ 7	60	8.9	60	8.8	60	8.9	60	8.9
Child age								
1–3	223	32.9	238	35	212	31.3	239	35.4
4–6	281	41.4	294	43.2	282	41.6	285	42.2
7–9	174	25.7	148	21.8	184	27.1	151	22.4
School grade of a child								
0	527	77.7	486	71.5	406	59.9	428	63.4
≥ 1	151	22.3	194	28.5	272	40.1	247	36.6
Sex of a child								
Male	313	46.2	322	47.4	312	46	321	47.6
Female	365	53.8	358	52.6	366	54	354	52.4

### Water supply, hand and face washing habits

The availability and use of water in the study areas were compared between baseline and after WaSH interventions among intervention and non-intervention woredas. The results showed that except for the time required to fetch water (*P*>0.05, MacNemar test), type of water source and face washing habits showed significant improvement after the intervention period in intervention woredas (P<0.05, MacNemar test). However, in non-intervention woredas, time required to fetch water showed significant improvement after the one-year follow up period (*P*<0.05, MacNemar test) (Table 2).

**Table 2:** Status of WaSH activities among intervention and non-intervention woredas across both surveys in North and South Wollo Zones, ANRS, Ethiopia, 2014/2015.

Activities		Intervention Woredas			Non-intervention woredas		
		Baseline (n = 678)	After intervention (n = 678)	*P* value	Baseline (n = 680)	After intervention (n = 675)	*P* value
Primary water source	Improved	525 (77.4%)	568 (83.8%)	≤0.001*	403 (59.3%)	410 (60.7%)	0.306
	Unimproved	153 (22.6%)	110 (16.2%)		277 (40.7%)	265 (39.3%)	
						
Amount of water used per person per day	≤ 20 liters	617 (91%)	420 (62%)	1.8	630 (93%)	515 (76%)	6.96
	>20 liters	61 (9%)	258 (38%)		50 (7%)	160 (24%)	
Time required to fetch water	≤30 minutes	431 (63.6%)	445 (65.6%)	0.434	265 (39%)	336 (49.8%)	≤0.001*
	>30 minutes	247 (36.4%)	233 (34.4%)		415 (61%)	339 (50.2%)	
						
Face washing habits	Once a day	639 (94.2%)	32 (4.7%)	≤0.001*	599 (88.1%)	581 (86.1%)	0.319
	Two or more times a day	39 (5.8%)	646 (95.3%)		81 (11.9%)	94 (13.9%)	
						
Washing clothes	Once a week	657 (97%)	658 (97%)	0.87	660 (97%)	662 (98%)	0.73
	Once a month	21 (3%)	20 (3%)		20 (3%)	13 (2%)	
						
Soap use during hand and face washing	Yes	197 (29%)	315 (46%)	0.6	102 (15%)	109 (16%)	3.8
	No	481 (71%)	363 (54%)		578 (85%)	566 (84%)	

MacNemar test

* = significant values (*P* value <0.05)

### Hygiene and sanitation

According to the results of the MacNemar analysis, presence of ocular and nasal discharges significantly decreased after the one year intervention period in the intervention woredas (P≤0.001, MacNemar test). In addition, number of households with latrine and hand washing container near latrine, were increased. After the one-year intervention period in intervention woredas (P<0.05, MacNemar test); while in the non-intervention woredas, presence of nasal discharge didn’t decrease after the intervention period (P>0.05). (Tables 3 and 4).

**Table 3:** Effect of hygiene and sanitation activities at baseline and follow up periods among intervention and non-intervention woredas of North and South Wollo zones, ANRS, Ethiopia, 2014/2015.

Activities		Intervention Woredas			Non-intervention woredas		
		Baseline (n = 678)	After intervention(n = 678)	*P* value	Baseline (n = 680)	After intervention(n = 675)	*P* value
Ocular discharge	Yes	134 (19.8%)	55 (8.1%)	≤0.001*	87 (12.8%)	112 (16.6%)	≤0.001*
	No	544 (80.2%)	623 (91.9%)		593 (87.2%)	563 (83.4%)	
						
Nasal discharge	Yes	167 (24.6%)	153 (22.6%)	≤0.001*	201 (29.6%)	196 (29%)	0.481
	No	511 (75.4%)	525 (77.4%)		479 (70.4%)	479 (71%)	
						
Access to improved latrine	Yes	314 (46.3%)	402 (59.3%)	≤0.001*	265 (39%)	320 (47.4%)	≤0.001*
	No	364 (53.7%)	276 (40.7%))		415 (61%)	355 (52.6%)	
						
Observable feces in the compound	Yes	170 (25%)	120 (18%)	1.71	86 (13%)	88 (13%)	1.262
	No	508 (75%)	558 (82%)		594 (87%)	587 (87%)	

MacNemar test

* = significant values (*P* value <0.05)

**Table 4:** Effect of sanitation activities at baseline and follow up periods among intervention and non-intervention woredas of North and South Wollo zones, ANRS, Ethiopia, 2014/2015.

Activities		Intervention Woredas			Non-intervention woredas		
		Baseline (n = 314)	After intervention (n = 407)	*P* value	Baseline (n = 265)	After intervention (n = 315)	*P* value
Frequency of latrine use	Always	250 (79.6%)	336 (82.6%)	0.008*	203 (76.6%)	258 (81.9%)	0.001*
	Sometimes	64 (20.4%	71 (17.4%)		62 (23.4%)	57 (18.1%)	
						
Availability of hand washing container near the latrine	Yes	26 (8.3%	140 (34.4%)	≤0.001*	30 (11.3%)	250 (79.4%)	0.012*
	No	288 (91.7%)	267 (65.6%)		235 (88.7%)	65(20.6%)	
						
Availability of water in the hand washing container	Yes	4 (15%)	25 (18%)	0.76	4 (13%)	10 (15%)	0.99
	No	22 (85%)	115 (82%)		26 (87%)	55 (85%)	

MacNemar test

* = significant values (*P* value <0.05)

### Prevalence of active trachoma

There was a significant reduction in the prevalence of active trachoma after one-year WaSH intervention in intervention woredas (P<0.05, MacNemar test). However, non-intervention woredas didn’t show significant reduction after one-year study period compared to the baseline survey (Table 5).

**Table 5:** Prevalence of active trachoma at baseline and after intervention in the intervention and non-intervention woredas, ANRS, Ethiopia, 2014/2015.

Stages of trachoma	Intervention Woredas				Non-intervention woredas			
	Baseline(n = 678)		After intervention (n = 678)	*P* value	Baseline (n = 680)		After intervention (n = 675)	*P* value
Active Trachoma	Yes	176 (26%)	124 (18.3%)	≤0.001*	Yes	118 (17.4%)	116 (17.2%)	0.727
	No	502 (74%)	554 (81.7%)		No	562 (82.6%)	559 (82.8%)	
Trachomatous inflammation-follicular	Yes	156 (23%)	105 (15.5%)	≤0.001*	Yes	92 (13.5%)	91 (13.5%)	1.000
	No	522 (77%)	573 (84.5%)		No	588 (86.5%)	584 (86.5%)	
Trachomatous inflammation-intense	Yes	33 (4.9%)	26 (3.8%)	0.016*	Yes	32 (4.7%)	29 (4.3%)	0.375
	No	645 (95.1)	652 (96.2%)		No	648 (95.3%)	646 (95.7%)	

MacNemar test

* = significant values (*P* value <0.05)

### Discussion

The current quasi-experimental study in North and South Wollo Zones of ANRS, Ethiopia measured the effect of WaSH intervention after one-year follow up period. The follow up survey was carried out using the same methods in the same communities as the baseline survey. Our study showed a remarkable improvement in the status and practice of water supply, sanitation and hygiene situation after the intervention period in the intervention woredas as compared to the non-intervention ones. Households from the intervention woredas had better access to improved water sources and they washed their hands and face more than two times a day. The frequency of latrine use was improved significantly in the intervention woredas than the non-intervention ones and more households were free from open defecation.

Compared to the non-intervention woredas, presence of ocular and nasal discharges significantly decreased after the intervention period in the intervention woredas. Thus, many children were observed to have clean faces after the intervention. This might be due to the coordinated approach of ORDA and ATCP in the community that aimed at developing water schemes, latrines as well as health education activities on hygiene and sanitation at community as well as school levels. The establishment of Anti-Trachoma School Clubs in primary schools may also have a contribution for the improvement of the current hygiene and sanitation practices of the community at school and community level. There were sanitation corners and trachoma ambassadors in schools. These ambassadors were checking the personal hygiene of students, and when they observed poor personal hygiene, the students were taken to the sanitation corner so that he/she could wash his/her face and get education about personal hygiene. This approach may have contributed for the improvement of the current hygiene and sanitation situation in the community.

The prevalence of active trachoma observed among children in both surveys among intervention and non-intervention woredas in the current study was above the 2020 global trachoma elimination target as set by the WHO [9]. However, after one-year WaSH intervention, there was a significant reduction in the prevalence of active trachoma in all communities in the intervention woredas, but not in the non-intervention woredas. This might be related to the effectiveness of the trachoma control program conducted in the intervention woredas,

Several factors may have contributed to improved trachoma control during the one year ATCP intervention period. Thirty-seven water supply schemes were developed by ORDA in addition to the development of improved water supply schemes by the Regional Water, Irrigation and Energy Development Bureau in both intervention and non-intervention woredas. Similarly, the community sensitization by ATCP workers has improved the construction and utilization of latrines. Therefore, these activities may have contributed to the significant reduction of the prevalence of active trachoma in the intervention woredas as compared to the non-intervention ones.

Our finding is consistent with other study results, for instance, a systematic review and meta-analysis done in October 2013 revealed a strong association between improved WaSH conditions and reduced trachoma [18, 19]. A study conducted in December 2012 also confirmed the significant contribution of WaSH implementation for sustained control, elimination, or eradication of trachoma [6]. A population-based survey conducted in Malawi showed that sustained reductions in active trachoma could be achieved without community-based antibiotic distribution through health, water and hygiene programs [20, 21]. In line with the current study, a review of 19 studies selected from different parts of the world showed the sustainable role of hygiene and environmental improvements on trachoma control in a population where trachoma is endemic rather than the short-term impact of antibiotic treatment [22–24]. The absence of significant reduction in the prevalence of active trachoma observed in the non-intervention woredas of the current study also strengthens the effect of WaSH intervention on trachoma control.

Overall, endemic countries strategy to control and eliminate trachoma needs improved access to, and use of, water, sanitation and hygiene. Otherwise, the administration of antibiotics and correction of trichiasis cases alone to at-risk populations may not eliminate trachoma, due to its high re-occurrence nature [25]. Therefore, there should be a collaborative programming on WaSH practice to control and eliminate trachoma from its endemic areas.

This study has both strengths and limitations. The use of large sample size, the presence of control woredas for comparison purpose and the internal consistency of our data indicate strength of the study. Limitations of the study are that the estimation of household fetched water volume per day and time taken to fetch water were merely based on respondents’ response to the interviewer questions, which may be uncertain. The errors due to this were minimized by asking different individuals from the households to answer the same question and taking the average value by the interviewer. In addition, the overlap in the use of facilities and education regarding hygiene practices may affect the internal validity of the study

## Conclusions

The study showed a statistically significant reduction of active trachoma prevalence in the intervention woredas compared to the base line study. The non-intervention woredas did not show significant reduction of prevalence after the intervention period compared to the baseline survey. The finding also supports the association between active trachoma and WaSH intervention. It is therefore likely that the observed reductions in active trachoma prevalence after the intervention period may be linked to the effect WaSH interventions. However, even though we observed significant reduction of prevalence after the one-year intervention period, the magnitude of the trachoma prevalence in the study area is still very high. Therefore, to achieve trachoma elimination target by the year 2020 as set by the WHO, continued WaSH interventions and periodic monitoring, evaluation and reporting of the impact of WaSH on active trachoma is warranted in the study area.

## Supporting information

S1 Checklist STROBE checklist. (DOC)

S1 Dataset Baseline and intervention data’s for both intervention and non-intervention woredas. (RAR)
