# Supplementary material for: Effect of water, sanitation and hygiene interventions on active trachoma in North and South Wollo zones of Amhara Region, Ethiopia: A Quasi-experimental study
Source: PLoS Negl Trop Dis. 2017 Nov 10;11(11):e0006080. doi: 10.1371/journal.pntd.0006080 (PMC5699846; doi:10.1371/journal.pntd.0006080)
Supplement: S1 Checklist — (DOC) [file pntd.0006080.s001.doc]

STROBE checklist

|  | Item No | Recommendation |
| --- | --- | --- |
| **Title and abstract** | 1 | (a) Effect of Water, Sanitation and Hygiene interventions on active trachoma in North and South Wollo Zones of Amhara Region, Ethiopia: A Quasi-experimental study |
|  |  | (b)A community based quasi-experimental study was conducted from February 2014 to July 2015 among children aged 1-8 years at baseline and among one year older same children after intervention. A four-stage random cluster-sampling technique was employed to select study participants. From each selected household, one child was clinically assessed for active trachoma. Structured questionnaire was used to collect socio demographic and behavioural data. MacNemar test was applied to compare the prevalence of active trachoma between baseline and after the intervention period at both intervention and non-intervention study areas.  The prevalence of active trachoma was reduced from baseline prevalence of 26% to 18% after a one-year intervention period in the intervention woredas (*P*≤0.001). MacNemar test result showed significant reduction of active trachoma prevalence after the intervention period in the intervention woredas compared to the non-intervention woredas (P≤0.001). Water, sanitation and hygiene related activities were significantly improved after the intervention period in the intervention woredas (P<0.05). |
| Introduction | | |
| Background/rationale | 2 | Trachoma, chronic kerato conjunctivitis, is caused by repeated infection with *Chlamydia trachomatis* bacterium. It is hyper endemic in many rural areas of Ethiopia. Trachoma can be prevented through proper hygiene, increased facial cleanliness and sanitation. F and E components of the SAFE strategy is one of the most important indicators for effective prevention and eventual elimination of trachoma. Unless improved WASH is attained and maintained in a sustainable manner the prevalence of trachoma infection in the affected communities will remain high causing visual impairment and blindness. |
| Objectives | 3 | -To measure the prevalence of active trachoma  -To measure the effect of WaSH program implementation on active trachoma burden after one-year intervention period in the same woredas where the base line survey was conducted.  -The prevalence of active trachoma was less among children from households with better and WaSH facilities used compared to those with less WaSH facilities and not used it properly |
| Methods | | |
| Study design | 4 | This was a quasi-experimental study |
| Setting | 5 | The study was conducted in North and South Wollo Zones of ANRS, Ethiopia. The WHO recommended WaSH program is being implemented in selected woredas of ANRS. Considerable proportion of woredas in North and South Wollo Zones are included in this initiative. Similarly, the Amhara Trachoma Control Program (ATCP) has been operating in Dessie Zuriya and Raya Kobo woredas of North and South Wollo zones. The program’s overall objective complements the WHO campaign for the Global Elimination of Blinding Trachoma by the year 2020 and adopts multi-sectoral comprehensive approach. Provision of water as well as education of the community on hygiene and sanitation has been the focuses areas of the program. The baseline survey was conducted from February 2014 to April 2014. After the one year intervention, the follow up survey took place from May 2015 to July 2015. |
| Participants | 6 | Participants were a random sample of children aged 1-8 years at baseline and 1-9 years at follow up. Caregivers of the diagnosed children were also the participants of the study. |
|  |  |  |
| Variables | 7 | The people in intervention areas were exposed to WaSH intervention. The prevalence of active trachoma was reduced after the exposure. The overlap in the use of facilities and education regarding hygiene practices were the potential confounders. |
| Data sources/ measurement | 8* | The data sources were selected children and their caregivers |
| Bias | 9 | The data collectors were informants who have recently experienced in trachoma diagnosis |
| Study size | 10 | It was calculated with double population proportion formula using Epi-Info software package by considering a 95% confidence interval (Z_α/2_ = 1.96), 5% type I error, 80% power, design effect of 1.5, 52% prevalence of active trachoma from a previous study, a 42% proportion of active trachoma at some future date such that the quantity of (p_2_-p_1_) would be the size of the magnitude of change (10% difference), and a 10% non-response rate |
| Quantitative variables | 11 | It was analysed by recoding it as necessary and by grouping it to create a new categorical variable. |
| Statistical methods | 12 | (*a*) Comparison of the control and intervention groups using MacNemar test |
|  |  | (*b*) collinearity were done |
|  |  | (d) It was treated with the 10% non-response rate given during the sample size calculation |
| Results | | |
| Participants | 13* | At baseline 1358 children  At follow up 1353 due to lost to follow up |
|  |  | (b) absence of the study participants during the follow up data collection |
| Descriptive data | 14* | (a) Characteristics of study participants have been found on page 14-15 of the manuscript |
|  |  | (b) Indicate number of participants with missing data for each variable of interest |
|  |  | (c) the overall follow up period was one year |
| Outcome data | 15* | See Table 5 in the manuscript |
| Main results | 16 | There was a significant reduction in the prevalence of active trachoma after a one-year WaSH intervention in intervention woredas (P<0.05, MacNemar test). |
| Other analyses | 17 | ------- |
| Discussion | | |
| Key results | 18 | The prevalence of active trachoma observed among children in both surveys among intervention and non-intervention woredas in the current study was above the 2020 global trachoma elimination target as set by the WHO. However, after one-year WaSH intervention, there was a significant reduction in the prevalence of active trachoma in all communities in the intervention woredas, but not in the non-intervention woredas. |
| Limitations | 19 | Limitations of the study are that the estimation of household fetched water volume per day and time taken to fetch water were merely based on respondents’ response to the interviewer questions, which may be uncertain. In addition, the overlap in the use of facilities and education regarding hygiene practices may affect the internal validity of the study |
| Interpretation | 20 | Several factors may have contributed to improved trachoma control during the one year ATCP intervention period, 37 water supply schemes were developed by ORDA in addition to the development of improved water supply schemes by the Regional Water, Irrigation and Energy Development Bureau in both intervention and non-intervention a woredas. Similarly, the community sensitization by ATCP workers has improved the construction and utilization of latrines. Therefore, these activities may have contributed to the significant reduction of the prevalence of active trachoma in the intervention woredas as compared to the non-intervention ones. |
| Generalisability | 21 | To achieve trachoma elimination target by the year 2020 as set by the WHO, continued WaSH interventions and periodic monitoring, evaluation and reporting of the impact of WaSH on active trachoma is warranted in endemic areas. |
| Other information | | |
| Funding | 22 | The Ethiopian Institute of Water Resources provided funding for this study. The funders had no responsibility in the study design, data collection, data analysis, interpretation of the data, and preparation of the manuscript for publication |

*Give information separately for exposed and unexposed groups.

**Note:** An Explanation and Elaboration article discusses each checklist item and gives methodological background and published examples of transparent reporting. The STROBE checklist is best used in conjunction with this article (freely available on the Web sites of PLoS Medicine at http://www.plosmedicine.org/, Annals of Internal Medicine at http://www.annals.org/, and Epidemiology at http://www.epidem.com/). Information on the STROBE Initiative is available at http://www.strobe-statement.org.
